# Regulation of Orai1/STIM1 mediated I_CRAC_ by intracellular pH

**DOI:** 10.1038/s41598-017-06371-0

**Published:** 2017-08-29

**Authors:** D. Gavriliouk, N. R. Scrimgeour, S. Grigoryev, L. Ma, F. H. Zhou, G. J. Barritt, G. Y. Rychkov

**Affiliations:** 1 School of Medical Sciences, University of Adelaide, Adelaide, South Australia 5005 Australia; 2 School of Medicine, University of Adelaide, and South Australian Health and Medical Research Institute, Adelaide, South Australia 5005 Australia; 3 School of Medicine, Flinders University of South Australia, Bedford Park, South Australia 5065 Australia; 4 Institute for Molecular Bioscience, Present Address: University of Queensland, Brisbane, QLD 4072 Australia

## Abstract

Ca^2+^ release activated Ca^2+^ (CRAC) channels composed of two cellular proteins, Ca^2+^-sensing stromal interaction molecule 1 (STIM1) and pore-forming Orai1, are the main mediators of the Ca^2+^ entry pathway activated in response to depletion of intracellular Ca^2+^ stores. Previously it has been shown that the amplitude of CRAC current (I_CRAC_) strongly depends on extracellular and intracellular pH. Here we investigate the intracellular pH (pH_i_) dependence of I_CRAC_ mediated by Orai1 and STIM1ectopically expressed in HEK293 cells. The results indicate that pH_i_ affects not only the amplitude of the current, but also Ca^2+^ dependent gating of CRAC channels. Intracellular acidification changes the kinetics of I_CRAC_, introducing prominent re-activation component in the currents recorded in response to voltage steps to strongly negative potentials. I_CRAC_ with similar kinetics can be observed at normal pH_i_ if the expression levels of Orai1 are increased, relative to the expression levels of STIM1. Mutations in the STIM1 inactivation domain significantly diminish the dependence of I_CRAC_ kinetics on pH_i_, but have no effect on pH_i_ dependence of I_CRAC_ amplitude, implying that more than one mechanism is involved in CRAC channel regulation by intracellular pH.

## Introduction

Under normal physiological conditions extracellular pH (pH_o_) in healthy tissues is maintained within a narrow range between 7.3 and 7.4, while intracellular pH (pH_i_) is kept between 7.1–7.2^1^. In exercising muscle both extracellular and intracellular pH can drop as low as 6.9 and 6.7 respectively^2^. In cancerous tumours and wounded tissue, pH variations from the normal values can be even more extreme^3–6^. Dysregulated pH is one of the hallmarks of cancer progression, whereas pH of a wound can be used as a predictor of wound healing outcomes^4–7^. Activity of almost ubiquitously expressed Ca^2+^ release activated Ca^2+^ (CRAC) channels, formed by Orai1 and STIM1 proteins, has been shown to strongly depend on both extracellular and intracellular pH^8–10^. Considering that CRAC channels have an important role in regulation of immune response, skeletal muscle function and in cancer progression, their dependence on pH is likely to have some physiological significance.

Extracellular acidification inhibits, whereas alkalinisation increases CRAC current (I_CRAC_) amplitude with pK_a_ of about 8. This has been consistently shown in several publications using both, heterologous expression of Orai1 and STIM1 and cells expressing endogenous I_CRAC_^8–11^. The dependence of I_CRAC_ on extracellular pH in the presence of extracellular Ca^2+^ is mediated by E106 in the Orai1 pore, with some contribution from nearby D110 and D112 in the first extracellular loop^8, 9^. In the absence of Ca^2+^, Na^+^ permeability seems to be affected by protonation of E190 residue in the Orai1 pore^10^. The mechanism of I_CRAC_ dependence on intracellular pH is less well understood. Intracellular acidification has been shown to inhibit both, endogenous I_CRAC_ in different types of cells, and I_CRAC_ mediated by Orai1 and STIM1 heterologously expressed in HEK293 cells^8, 10, 12^. However, intracellular alkalinisation strongly enhanced the amplitude of Orai1/STIM1 mediated I_CRAC_ in some, but not all studies^10^, whereas the amplitude of endogenous I_CRAC_ in RBL cells and Jurkat T lymphocytes was not affected by pH_i_ rise from 7.4 to 8.4^8, 12^.

Theoretically, there are two main mechanisms that may mediate the dependence of I_CRAC_ function on pH_i._ Protonation/deprotonation of specific residues in Orai1 may affect conductance through the Orai1 pore, and/or protonation/deprotonation of specific residues in Orai1 and/or STIM1 may affect their interaction. The evidence obtained thus far cannot exclude either of these possibilities. Intracellular acidification has been shown to functionally uncouple STIM1 and Orai1 without causing a complete dissociation of STIM1/Orai1 complex, suggesting that pH_i_ affects STIM1/Orai1 interaction^13^. Furthermore, mutation of His155 in the intracellular loop of Orai1 to phenylalanine (H155F) abolishes the effect of alkalinisation on I_CRAC_ and diminishes I_CRAC_ inhibition caused by acidification of pH_i_^10^. The Orai1 region containing H155 has previously been implicated in fast Ca^2+^-dependent inactivation (FCDI) of I_CRAC_, and therefore may be involved in STIM1/Orai1 interactions^14^.

In this work, we investigated pH_i_ dependence of I_CRAC_ mediated by WT Orai1 and WT or mutated STIM1 ectopically expressed in HEK293 cells at two Orai1:STIM1 expression ratios. Using cells expressing WT Orai1 and WT STIM1 we confirmed that I_CRAC_ amplitude strongly depends on pH_i_ and showed that intracellular acidification introduces a strong re-activation component in I_CRAC_ traces recorded in response to voltage steps between −80 mV and −140 mV. (In this study, term “re-activation” is used exclusively in relation to slow increase of I_CRAC_ amplitude during voltage steps from 0 mV to potentials between −80 and −140 mV, and is opposite of FCDI.) As shown previously, this I_CRAC_ re-activation could also be observed at normal pH_i_, but only in the cells that were transfected with higher amounts of Orai1 cDNA, relative to STIM1^9, 15, 16^. To investigate whether there is any overlap between the mechanisms that regulate dependence of I_CRAC_ on pH_i_ and pH_o_, we used E106D Orai1 mutant. Glutamate 106 in the selectivity centre of Orai1 pore was previously shown to mediate I_CRAC_ dependence on pH_o_^9^. In further search for the potential protonation sites that may be responsible for I_CRAC_ dependence on pH_i_, we evaluated EE482/483AA and DD475/476AA double mutations within STIM1 inactivation domain (ID_STIM_). Considering that ID_STIM_ is highly negatively charged and is indispensable for Ca^2+^ dependent inactivation of I_CRAC_^17, 18^, it is logical to hypothesise that it is involved in pH_i_ sensitivity of CRAC channel.

## Results

### Intracellular pH affects CRAC channel gating

**Figure 1:**
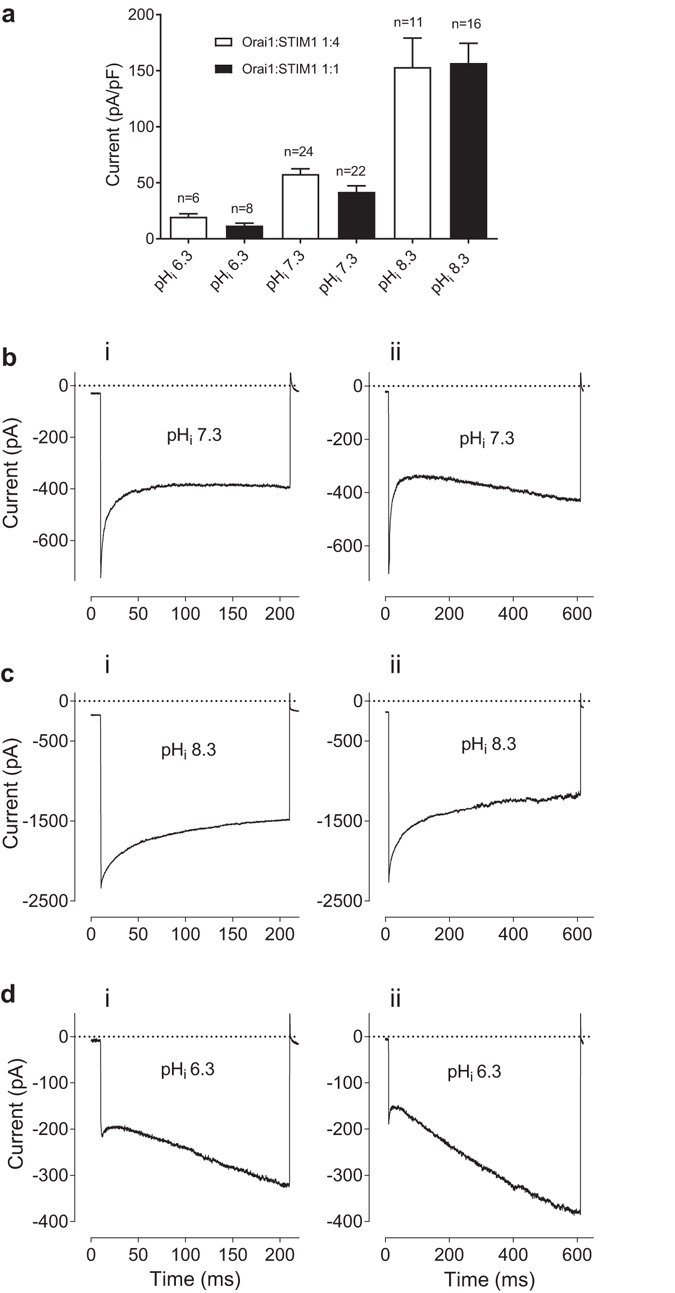
The dependence of I_CRAC_ amplitude and kinetics on pH_i_. (**a**) Current density at −100 mV, obtained from instantaneous I–V plots in response to 100 ms ramps between −120 and 120 mV recorded after a complete development of I_CRAC_, at indicated pH_i_ and Orai1:STIM1 transfection ratios. The amplitude of I_CRAC_ at pH_i_ 7.3 was significantly different from the amplitudes at pH_i_ 6.3 and 7.3 at both transfection ratios (P < 0.001; unpaired *t*-test). The amplitudes of I_CRAC_ at 1Orai1:1STIM1 transfection ratio was significantly smaller that the amplitudes at 1Orai1:4STIM1 ratio at pH_i_ 6.3 and 7.3 (P < 0.03; unpaired *t*-test), but not at pH_i_ 8.3. (**b**,**c** and **d**) The examples of WT I_CRAC_ traces at pH_i_ 7.3, 8.3 and 6.3, correspondingly. Currents were recorded in HEK293T cells transfected with Orai1 and STIM1 at 1:4 ratio, in response to 200 ms (i) and 600 ms (ii) voltage steps from 0 mV holding potential to −120 mV.

To investigate I_CRAC_ dependence on pH_i_, the currents were recorded using pipette solutions with pH_i_ adjusted to 6.3, 7.3 or 8.3. Previously it has been shown that the current amplitude, fast Ca^2+^ dependent inactivation (FCDI), re-activation, potentiation by 2-APB, and selectivity of CRAC channels for divalent cations strongly depend on the relative amounts of Orai1 and STIM1 proteins in the cell^15, 16^. It is possible that other properties of I_CRAC_, including pH dependence, are also influenced by the Orai1:STIM1 expression ratios. Therefore, we investigated the effects of pH_i_ on I_CRAC_ at two transfection conditions. To achieve different expression ratios, HEK293T cells were transfected with Orai1- and STIM1-containing plasmids at either 1:4 or 1:1 molar ratios. For both transfection conditions, the amplitude of I_CRAC_ exhibited strong dependence on pH_i_. I_CRAC_ was smaller at pH_i_ 6.3 and larger at pH_i_ 8.3, compared to pH_i_ 7.3 (Fig. 1a). Consistent with previous publications, at pH_i_ 7.3 and 6.3 cells transfected with higher relative amount of STIM1 (1 Orai1: 4 STIM1 ratio) produced larger I_CRAC_, compared to cells transfected with equal amounts of STIM1 and Orai1 (1 Orai1: 1 STIM1 ratio; Fig. 1a). However, the effect of the relative expression ratio on the current amplitude, at least within the employed transfection range (1:4 and 1:1), was absent when pH_i_ was raised to 8.3 (Fig. 1a). Majority of cells at pH_i_ 7.3 produced I_CRAC_ with noticeable FCDI at potentials between −80 and −140 mV when transfected with 1 Orai1: 4 STIM1 ratio (Fig. 1b,i). Some re-activation was also evident with longer pulses (Fig. 1b,ii). It was observed that raising pH_i_ to 8.3 resulted in elimination of visible signs of the re-activation component, even with longer pulses. However, the extent of FCDI of the currents recorded in response to 200 ms pulses at pH_i_ 8.3 was also reduced, compared to pH_i_ 7.3, and the time course of inactivation was significantly slower (*c*.*f*. Fig. 1ci and bi). In contrast, lowering pH_i_ to 6.3 produced I_CRAC_ with pronounced re-activation at potentials between −80 and −140 mV and no visible FCDI (Fig. 1d).

**Figure 2:**
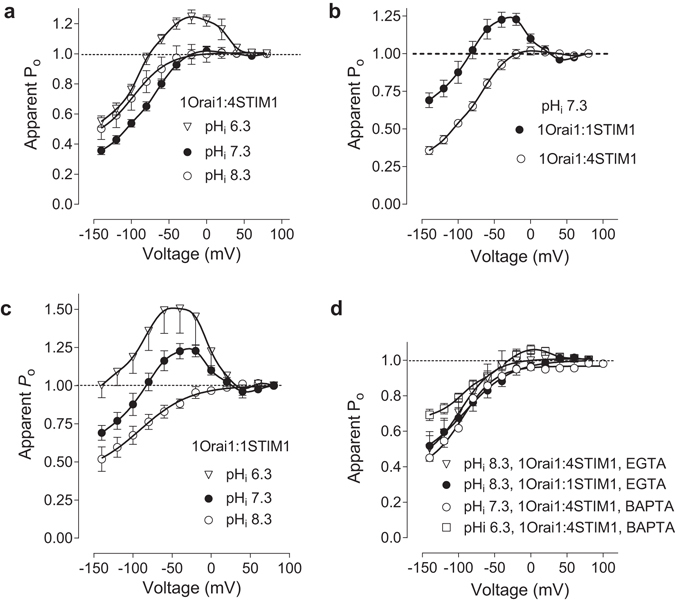
The dependence of WT I_CRAC_ apparent *P*_o_ on pH_i_. (**a**) Apparent *P*_*o*_ curves were obtained using tail currents as described in Methods. HEK293T cells were transfected with Orai1 and STIM1 at 1:4 ratio and I_CRAC_ was recorded at pH_i_ 6.3 (clear triangles, n = 6), 7.3 (filled circles, n = 16) and 8.3 (clear circles, n = 7). (**b**) The comparison of *P*_o_ curves obtained at Orai1:STIM1 transfection ratios 1:1 (filled circles, n = 9) and 1:4 (clear circles, n = 16) at pH_i_ 7.3. (**c**) Apparent *P*_o_ curves obtained from tail currents recorded in cells transfected with Orai1 and STIM1 at 1:1 ratio. I_CRAC_ was recorded at pH_i_ 6.3 (clear triangles, n = 4), 7.3 (filled circles, n = 9) and 8.3 (clear circles, n = 8). Data on panels (**a**,**b** and **c**) were obtained with pipette solution containing EGTA. (**d**) The effect of BAPTA on *P*_o_. Apparent *P*_o_ curves were obtained using cells transfected with Orai1 and STIM1 at 1:4 transfection ratio and BAPTA in the pipette solution at pH_i_ 7.3 (clear circles) and 6.3 (clear squares). For comparison, *P*_o_ curves obtained using cells transfected with Orai1 and STIM1 at either 1:4 (clear triangles) or 1:1 (filled circles) transfection ratio and EGTA in the pipette solution at pH_i_ 8.3 are shown.

To compare I_CRAC_ Ca^2+^ dependent gating (FCDI and re-activation) under different conditions and a range of membrane potentials, we used the amplitudes of tail-currents obtained at −100 mV after voltage steps between −140 and +80 mV, normalised to the amplitude of the tail current after a step to +80 mV (see Methods)^9^. The resulting data were used to construct apparent *P*_o_ curves^9^. I_CRAC_ that exhibited FCDI and little or no re-activation produced apparent *P*_o_ data that could be fitted with a standard Boltzmann equation (eq. 1), whereas I_CRAC_ with pronounced re-activation exhibited bell-shaped *P*_o_ curves which could not be fitted with a single Boltzmann function (Fig. 2a). At the 1Orai1:4STIM1 transfection ratio FCDI was more pronounced at pH_i_ 7.3 than at pH_i_ 8.3. At pH_i_ 6.3, the *P*_o_ curve was bell-shaped with a maximum at −20 mV, which was expected, considering the presence of re-activation. However, despite the apparent absence of FCDI in current traces recorded at pH_i_ 6.3 (Fig. 1d), the FCDI was still present, and the extent of it, relative to the maximum *P*_o_, was similar to that of I_CRAC_ recorded at pH_i_ 7.3 (Fig. 2a).

### Larger I_CRAC_ amplitude at alkaline pH_i_ is due to pH dependence of EGTA

Phenomenologically, the effect of intracellular acidification on the I_CRAC_ kinetics and *P*_o_ (Figs 1 and 2) was similar to the effect of increasing Orai1 expression relative to STIM1^16^. I_CRAC_ recorded at pH_i_ 7.3 in the cells transfected with 1Orai1:1STIM1 ratio showed strong re-activation during voltage steps from 0 mV to −120 mV and produced bell-shaped *P*_o_ curve, which looked similar to the *P*_o_ curve obtained at pH_i_ 6.3 with 1 Orai1: 4 STIM1 transfection ratio (*c*.*f*. Fig. 2a and b). Lowering pH_i_ to 6.3 in cells transfected with 1 Orai1: 1 STIM1 ratio further increased the re-activation (Fig. 2c). In contrast, rising pH_i_ to 8.3 virtually eliminated current re-activation (Fig. 2c). The apparent *P*_o_ curves obtained at pH_i_ 8.3 in cells transfected with 1:1 and 1:4 Orai1:STIM1 ratios were almost identical between two transfection conditions (Fig. 2d). The observed changes in the kinetics and the extent of I_CRAC_ FCDI induced by raising pH_i_ to 8.3 (Fig. 1c) are similar to those caused by replacing EGTA with BAPTA at pH_i_ 7.3^9, 19, 20^. Due to its’ ability to bind Ca^2+^ faster than EGTA, BAPTA is believed to reduce Ca^2+^ concentration at the intracellular mouth of CRAC channels, thus slowing down and reducing FCDI^19, 20^. The apparent *P*_o_ curve obtained at pH_i_ 7.3 using cells transfected with 1 Orai1: 4 STIM1 ratio and BAPTA as Ca^2+^ buffer, was virtually identical to *P*_o_ curves obtained at pH_i_ 8.3 and EGTA in the pipette solution (Fig. 2d). Using BAPTA in the internal solution instead of EGTA with pH_i_ 6.3 also decreased I_CRAC_ re-activation at negative potentials and therefore reduced positive apparent *P*_o_ (Fig. 2d).

**Figure 3:**
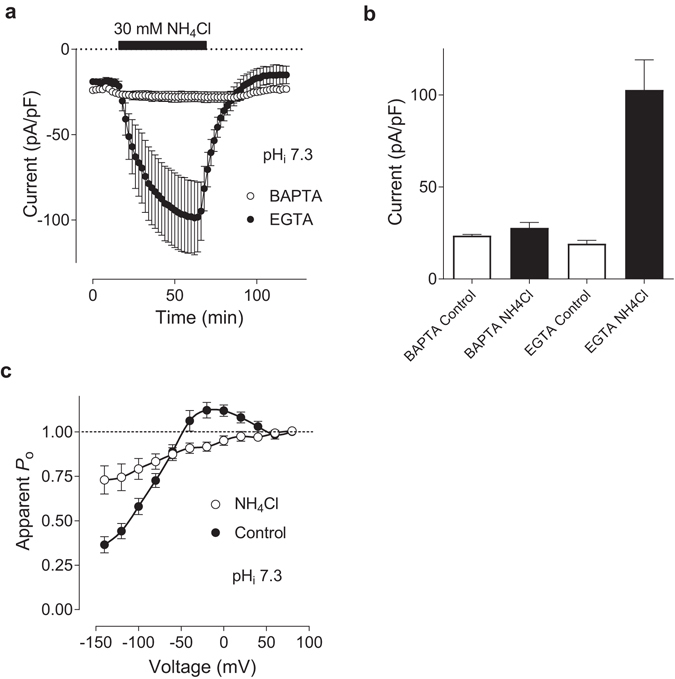
I_CRAC_ potentiation by intracellular alkalinisation depends on intracellular Ca^2+^ buffer. (**a**,**b**) The effect of 30 mM NH_4_Cl application to the bath on the amplitude of I_CRAC_. Each point on panel ***a*** represents I_CRAC_ amplitude at −100 mV obtained from 100 ms voltage ramps between −120 and 120 mV, applied every 2 s. WT I_CRAC_ was recorded in cells transfected with Orai1 and STIM1 at 1:4 ratio using either 10 mM BAPTA (clear circles) or 10 mM EGTA (filled circles) in the pipette solution (n = 5 for each condition), pH_i_ 7.3. (**c**) Apparent *P*_*o*_ curves obtained before (filled circles) and after (clear circles) application of NH_4_Cl to the bath (n = 5).

To investigate whether intracellular Ca^2+^ buffer contributes to the dependence of I_CRAC_ on pH_i_, we used extracellular application of 30 mM NH_4_Cl, which is known to alkalinise pH_i_^13, 21^. Application of NH_4_Cl to the bath, when EGTA was used as Ca^2+^ buffer in the pipette solution, drastically increased the I_CRAC_ amplitude (Fig. 3a,b) and caused inhibition of both I_CRAC_ FCDI and re-activation (Fig. 3c), in agreement with the results obtained using pipette solution with EGTA and pH_i_ 8.3 (Figs 1a and 2a,c). In contrast, application of NH_4_Cl when BAPTA was used in the pipette solution instead of EGTA, had very little effect on I_CRAC_ amplitude (Fig. 3a,b).

### Is there any overlap between mechanisms regulating I_CRAC_ dependence on pH_o_ and pH_i_?

**Figure 4:**
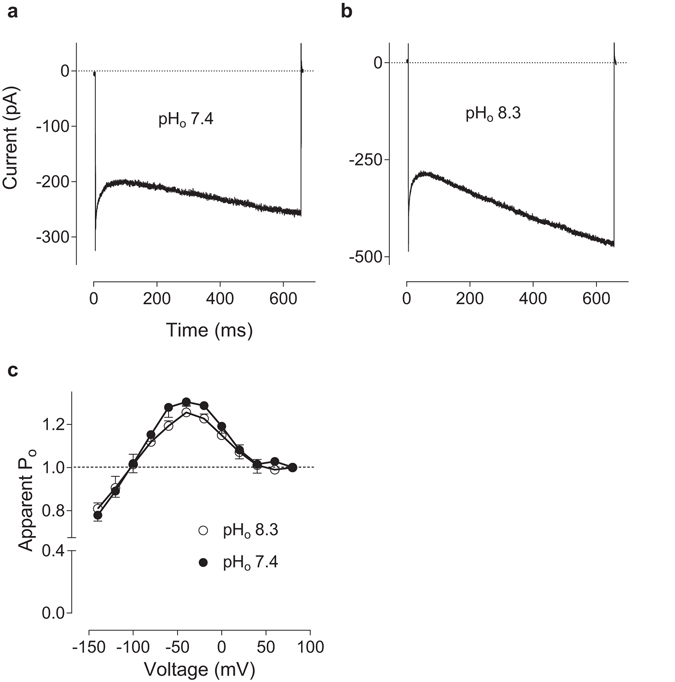
Extracellular alkalinisation has no effect on I_CRAC_ kinetics. (**a**,**b**) Examples of I_CRAC_ traces recorded in response to −120 mV steps in the bath solution of pH_o_ 7.4 and after changing pH_o_ to 8.3, correspondingly. (**c**) Apparent *P*_o_ curves at pH_o_ 7.4 (filled circles) and after changing pH_o_ to 8.3 (clear circles) (n = 4). HEK293T cells were transfected with Orai1 and STIM1 plasmids at 1:1 molar ratio. pH of the pipette solution was 7.3.

Previous investigations have shown that the amplitude of native I_CRAC_ in different cell types and I_CRAC_ mediated by heterologously expressed Orai1 and STIM1 strongly depends on extracellular pH^8–10, 12^. Superficially, the dependence of I_CRAC_ amplitude on pH_o_ looks similar to its dependence on pH_i_^8–10, 12^. However, possible reasons for similarities between pH_i_ and pH_o_ effects on I_CRAC_ have not been yet considered. Could changing pH_o_ affect pH_i_ in patch clamping experiments? To investigate this question, we used cells transfected with Orai1 and STIM1 at 1:1 molar ratio, which showed a very pronounced re-activation at negative potentials (Fig. 4a). Raising pH_i_ to 8.3 eliminates I_CRAC_ re-activation at negative potentials (Fig. 2b), and if raising pH_o_ results in a rise of pH_i_, one would expect a reduction of current re-activation. The results show that increasing pH_o_ from 7.4 to 8.3 does not reduce I_CRAC_ re-activation and has no effect on the *P*_o_ curve (Fig. 4). Therefore, it can be safely concluded that pH_i_ in these patch clamping experiments is not affected by changes in pH_o_.

**Figure 5:**
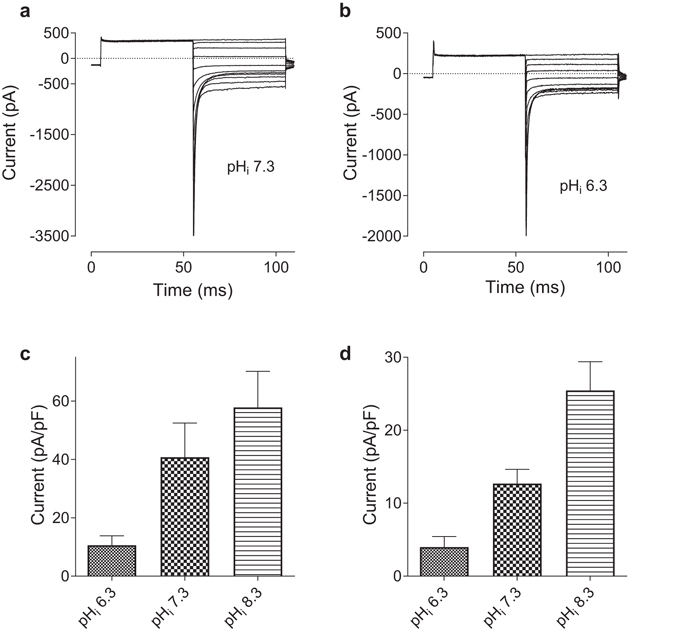
pH_i_ dependence of E106D-mediated I_CRAC_. (**a**,**b**) E106D Orai1 current traces were recorded in response to voltage steps ranging from −120 mV to 80 mV, in 20 mV increments, after a pre-pulse to 80 mV at pH_i_ 7.3, and pH_i_ 6.3, correspondingly. (**c**,**d**) The dependence of E106D Orai1 mediated I_CRAC_ amplitude on pH_i_ in the control bath solution (**c**), and in a bath solution with 140 mM NaCl replaced with 140 mM NMDGCl (**d**). HEK293T cells were transfected with E106D Orai1 and STIM1 plasmids at 1:4 molar ratio.

One of the main residues responsible for I_CRAC_ dependence on pH_o_ is Glu 106 in the Orai1 pore^9^. Could pH_i_ affect protonation of Glu 106 in the Orai1 pore? Despite the observation that I_CRAC_ kinetics is unaffected by pH_o_, it is possible that I_CRAC_ amplitude dependence on pH_i_ and pH_o_ is mediated by the same protonatable site in the pore. To investigate this possibility, we used an E106D Orai1 mutant. Previous studies have shown that the E106D Orai1 differs from WT Orai1 in several respects^9^. Firstly, it is less selective for Ca^2+^ and supports a significant Na^+^ conductance. Secondly, while E106D-mediated I_CRAC_ exhibits strong inactivation at negative potentials that looks similar to FCDI of WT I_CRAC_ (Fig. 5a, *cf*. Fig. 1b), it has a different underlying mechanism. The inactivation of E106D-mediated I_CRAC_ during steps to negative potentials is caused by Ca^2+^ block of Na^+^ permeation through the pore; it does not require interaction with ID_STIM_, and it is not affected by BAPTA or Orai1:STIM1 transfection ratios^9^. Finally, and importantly for this investigation, the Ca^2+^ dependent block of Na^+^ permeation through the E106D pore is strongly pH_o_ dependent, whereas the peak amplitude of I_CRAC_ mediated by E106D Orai1 is not influenced by pH_o_^9^. Changing the pipette solution pH revealed that the amplitude of E106D-mediated I_CRAC_ was pH_i_-dependent – the current was strongly inhibited by pH_i_ 6.3 and enhanced by pH_i_ 8.3, similarly to WT I_CRAC_ (Fig. 5c, *cf*. Fig. 1a). E106D-mediated I_CRAC_ recorded in the absence of Na^+^ in the bath solution, when Ca^2+^ was the only permeating cation, also showed pH_i_ dependence of the amplitude similar to that of WT I_CRAC_ (Fig. 5d). However, the kinetics and the extent of Ca^2+^ dependent block of Na^+^ permeation through E106D Orai1 was not appreciably affected by pH_i_ (Fig. 5b, *cf*. Figs 5a and 1d). If Asp 106 could be protonated from the intracellular side at low pH_i_, one would expect the changes in E106D-mediated I_CRAC_ to be similar to those induced by low pH_o_^9^, which was not the case. These results demonstrate that pH_i_ and pH_o_ affect I_CRAC_ through different mechanisms, and that Glu 106 which is located in the Orai1 pore does not mediate the pH_i_-dependence of I_CRAC_ amplitude.

### The effects of mutations in STIM inactivation domain on I_CRAC_ dependence on pH_i_

**Figure 6:**
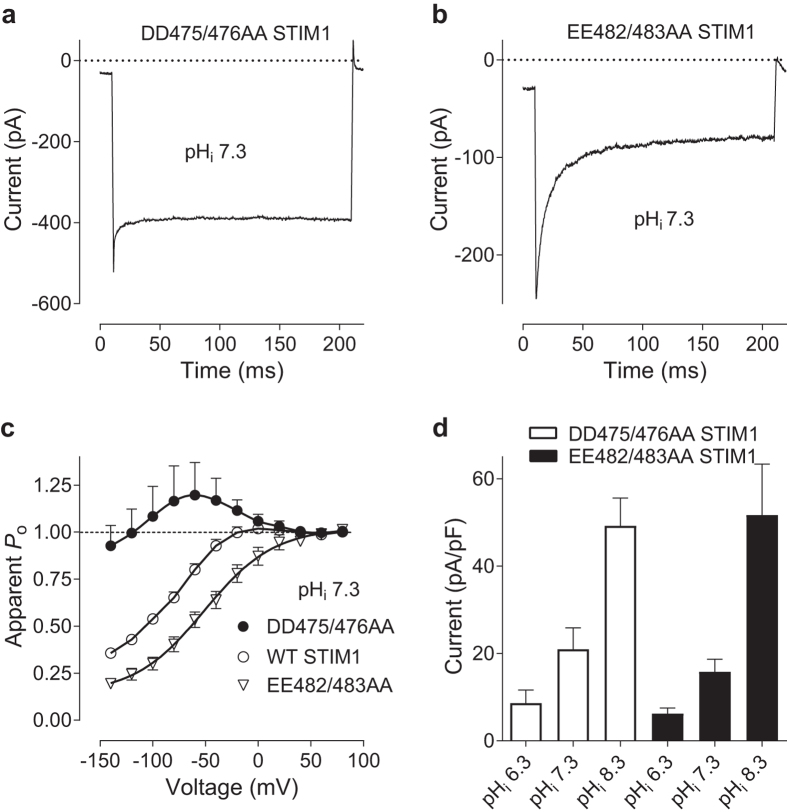
The dependence of I_CRAC_ amplitude, mediated by Orai1 and DD475/6AA and EE482/3AA STIM1 mutants, on pH_i_. (**a**,**b**) Examples of I_CRAC_ traces recorded in response to 200 ms voltage steps from 0 mV to −120 mV in cells transfected with Orai1 and either DD475/6AA STIM1 (**a**) or EE482/3AA STIM1 (**b**) at pH_i_ 7.3. (**c**) Apparent *P*_o_ curves obtained at pH_i_ 7.3 using cells transfected with Orai1 and DD475/6AA STIM1 (filled circles), Orai1 and WT STIM1 (clear circles), and Orai1 and EE482/3AA STIM1 (clear triangles), at 1:4 ratio. (**d**) ID_STIM_ mutants I_CRAC_ amplitude was measured at −100 mV from the responses to 100 ms voltage ramps from −120 to 120 mV, at indicated pH_i_. HEK293T cells were transfected with Orai1 and DD475/6AA STIM1 or EE482/3AA STIM1 plasmids at 1:4 ratio.

One of the domains within STIM1/Orai1 complex critically important for FCDI is located on STIM1 between residues 470 and 491 (ID_STIM_), C-terminal to CRAC activation domain (CAD)^17, 18^. Thus, neutralisation of Aspartate and Glutamate residues within a cluster of 7 negatively charged amino acids (475DDVDDMDEE483) in ID_STIM_ results in drastic changes in I_CRAC_ FCDI^18^. It is possible that protonation/deprotonation of some of these residues contribute to I_CRAC_ dependence on pH_i_. To investigate this possibility, we investigated pH_i_ dependence of two double mutants of STIM1, DD475/476AA, which produced I_CRAC_ with diminished FCDI (Fig. 6a,c), and EE482/483AA, which produced I_CRAC_ with enhanced FCDI (Fig. 6b,c)^17, 18^. Using pipette solutions with pH adjusted to 6.3, 7.3 or 8.3 we found that the amplitude of I_CRAC_ mediated by each of these STIM1 mutants co-expressed with WT Orai1 exhibited dependence on pH_i_ similar to that of WT I_CRAC_ (Fig. 6d; *cf*. Fig. 1a).

**Figure 7:**
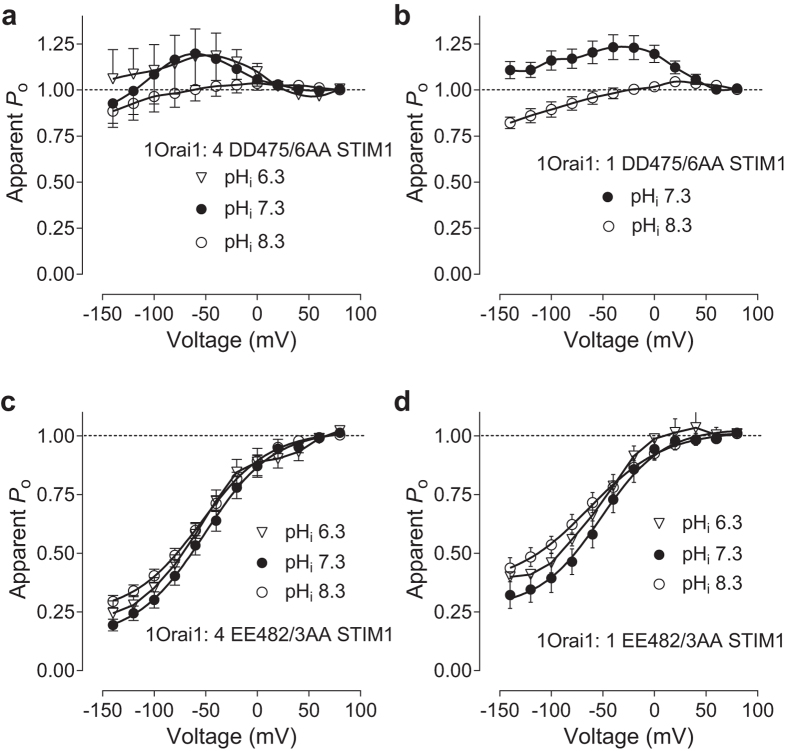
pH_i_ has no effects on the apparent *P*_o_ of I_CRAC_ mediated by Orai1 and DD475/6AA and EE482/3AA STIM1. (**a**,**b**) Apparent *P*_*o*_ curves obtained using cells transfected with Orai1 and DD475/6AA STIM1 at either 1:4 (**a**) or 1:1 (**b**) ratio and pH_i_ 6.3 (clear triangles), 7.3 (filled circles) and 8.3 (clear circles). (**c**,**d**) Apparent *P*_o_ curves obtained using cells transfected with Orai1 and EE482/3AA STIM1 at either 1:4 (**a**) or 1:1 (**b**) ratio and pH_i_ 6.3 (clear triangles), 7.3 (filled circles) and 8.3 (clear circles).

Next, we investigated the effects of DD475/476AA and EE482/483AA STIM1 mutations on the dependence of I_CRAC_ kinetics on pH_i_. At the transfection ratio of 1 Orai1: 4 STIM1 the apparent *P*_o_ for I_CRAC_ mediated by DD475/476AA-STIM1 mutant showed a weaker dependence on pH_i_, compared to WT I_CRAC_ (Fig. 7a; *cf*. Fig. 2a). Although pH_i_ 8.3 reduced the re-activation component (Fig. 7a), as it did in WT I_CRAC_ (Fig. 2a), pH_i_ 6.2 failed to induce a significant change in the apparent *P*_o_ of the Orai1/ DD475/476AA-STIM1 mediated current (Fig. 7a). Changing the transfection ratio to 1 Orai1: 1 DD475/476AA-STIM1 did not affect the apparent *P*_o_, or its dependence on pH_i_ (Fig. 7b). However, we were unable to obtain the apparent *P*_o_ curve at pH_i_ 6.3 as the amplitude of the current was too small for a reliable extraction of the data.

I_CRAC_ mediated by Orai1 and EE482/483AA-STIM1 mutant also exhibited a weaker dependence of the kinetics on pH_i_, compared to WT CRAC (Fig. 7c,d). At the transfection ratio 1 Orai1: 4 EE482/483AA-STIM1, lowering pH_i_ to 6.3 introduced a small re-activation component to the current (Fig. 7c). This can be seen on P_o_ curve as deviation from simple Boltzmann distribution, whereas increasing pH_i_ to 8.3 slightly reduced the extent of FCDI at negative potentials (Fig. 7c). At the transfection ratio 1:1, the changes in FCDI and re-activation induced by changes in pH_i_ were more pronounced than at the ratio 1:4 (Fig. 7d, *cf*. Fig. 7c), however, these changes were significantly smaller than those induced by pH_i_ changes in the WT I_CRAC_ (Fig. 2a,c; *cf*. Fig. 7c,d). Overall, DD475/476AA and EE482/483AA STIM1 double mutations significantly diminished the dependence of I_CRAC_ FCDI on pH_i_ and the relative Orai1/STIM1 expression ratio, without affecting pH_i_ dependence of I_CRAC_ amplitude.

**Figure 8:**
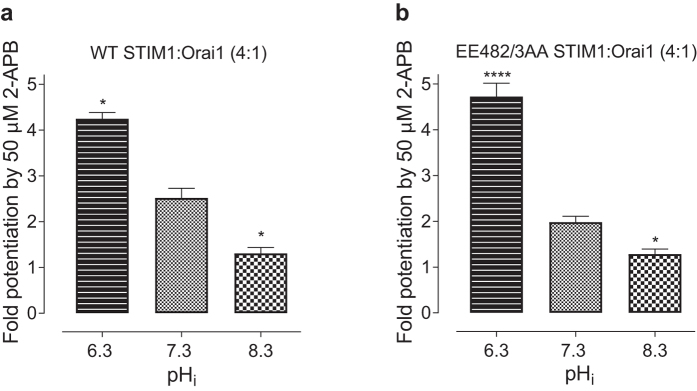
The pH_i_ dependence of I_CRAC_ potentiation by 2-APB. The Y-axis represents the ratio between the amplitudes of I_CRAC_ recorded immediately after and before application of 50 µM 2-APB to the bath. The I_CRAC_ amplitude was measured at −100 mV from the responses to 100 ms voltage ramps from −120 to 120 mV, applied every 2 seconds. HEK 293 T cells were transfected with WT STIM1 and Orai1 (**a**) or EE482/3AA STIM1and Orai1(**b**) at 4:1 ratio. pH of the pipette solution is indicated below the bars.

One of the distinctive properties of Orai1/STIM1 mediated I_CRAC_ is inhibition by high (over 100 µM) and potentiation by low (below 10 µM) concentrations of 2-APB, whereas application of intermediate concentrations of 2-APB (10–50 µM) cause transient potentiation of I_CRAC_ followed by inhibition^22^. Previously we have shown that the extent of I_CRAC_ potentiation by 2-APB depends on the relative expression levels of STIM1 and Orai1^16^. The higher the expression of Orai1, relative to STIM1, the stronger the potentiation^16^. Here we investigated whether potentiation of I_CRAC_ amplitude by 50 µM 2-APB is affected by pH_i_. The amplitude of I_CRAC_ in cells transfected with WT STIM1 and Orai1 at 4:1 ratio increased more than 4-fold at acidic pH_i_ of 6.3, but only 1.3-fold when pH_i_ was raised to 8.3, compared to a potentiation of 2.5-fold at pH 7.3 (Fig. 8a). Despite the lack of pH_i_ effect on FCDI and the apparent *P*_o_ of I_CRAC_ mediated by Orai1/EE482/483AA-STIM1, the dependence of 2-APB mediated potentiation of this mutant on pH_i_ remained unchanged, compared to WT I_CRAC_ (Fig. 8b).

## Discussion

The key findings of this paper can be summarised as follows – (i) pH_i_ regulates both, the amplitude of I_CRAC_ and Ca^2+^ dependent gating of CRAC channels; (ii) increase in I_CRAC_ amplitude in response to alkaline pH_i_ in the presence of EGTA in the pipette solution is a result of pH dependence of the Ca^2+^ buffering properties of EGTA, not the CRAC channel itself; (iii) Glutamate 106 in the selectivity centre of Orai1 pore, which mediates I_CRAC_ dependence on pH_o_, does not contribute to I_CRAC_ dependence on pH_i_; (iv) negatively charged residues in ID_STIM_ domain play a role in pH_i_ regulation of CRAC channel gating kinetics but not the amplitude of I_CRAC_. These data suggest that several mechanisms contribute to I_CRAC_ regulation by pH_i_.

It has been shown previously that increasing the amounts of Orai1 relative to STIM1 results in a smaller I_CRAC_ that exhibits re-activation at negative potentials which masks FCDI^15, 16^. The results presented here show that intracellular acidification has an effect on I_CRAC_ similar to that of increasing the relative amounts of Orai1 (or decreasing the relative amounts of STIM1). Comparable changes in I_CRAC_ kinetics and amplitude caused by intracellular acidification and increased Orai1:STIM1 ratio suggest that low pH_i_ reduces the affinity of STIM1 binding to Orai1, likely due to protonation of specific residues, which is equivalent to a reduction of available STIM1. This notion is supported by previous observations that acidification of cytoplasm due to hypoxia reduces FRET between Orai1and STIM1 and inhibits I_CRAC_^13^. In the study of Mancarella *et al*. (2012) the effect of hypoxia on I_CRAC_ could be mimicked by application of extracellular propionate, which lowers pH_i_, and reversed by application of NH_4_Cl, which raises pH_i_^13^. Intracellular acidification was shown to reduce FRET between STIM1-YFP and Orai1-CFP, but no change was observed in STIM1/Orai1 co-localisation in puncta^13^. These results suggested that pH_i_ affects STIM1/Orai1 functional coupling leading to channel opening, but not the interactions that trap STIM1 and Orai1 in puncta^13^. The pH dependent changes in I_CRAC_ kinetics reported here also point to the conclusion that intracellular acidification disrupts STIM1/Orai1 functional interactions.

Inhibition of I_CRAC_ by low pH_i_ has been demonstrated previously in several publications^8, 10, 12^. They all agree that I_CRAC_, both endogenous and mediated by ectopically expressed Orai1 and STIM1, is inhibited by approximately 70–90% at pH_i_ of around 6, compared to pH_i_ 7.3^8, 10, 12^. In contrast, the effects of alkalinisation of pH_i_ above 7.3 on I_CRAC_ are inconsistent between different studies^8, 10, 12^. The results of the present work suggest that the reason for the discrepancy is likely to be due to the type of intracellular Ca^2+^ buffer used. Studies employing BAPTA in the pipette did not find much increase in I_CRAC_ amplitude at higher pH_i_, whereas studies that used EGTA reported a significant potentiation of I_CRAC_ amplitude by alkalinisation^8, 10, 12^. Calculations using Maxchelator (http://maxchelator.stanford.edu/) indicate that Ca^2+^ buffering capacity of EGTA is highly pH dependent, and raising pH by one unit increases EGTA binding affinity to Ca^2+^ two orders in magnitude, changing K_d_ from 1.28 × 10^−7^ M at pH 7.3 to 1.4 × 10^−9^ M at pH 8.3, whereas pH dependence of Ca^2+^ buffering by BAPTA is weak.

The observations reported here which show strong increase in I_CRAC_ amplitude in response to NH_4_Cl application to the bath when EGTA is used in the pipette, and virtual absence of such effect when intracellular Ca^2+^ is buffered with BAPTA, suggest that the Ca^2+^ binding properties of EGTA play a significant part in I_CRAC_ pH_i_ dependence in the presence of EGTA, particularly, when pH_i_ rises above 7.5. The increase in I_CRAC_ amplitude at alkaline pH_i_ is likely to be due to stronger and faster Ca^2+^ binding by EGTA, rather than increase in pH_i_ *per se*. pH dependence of EGTA Ca^2+^ binding properties creates unwanted complications for the interpretation of the experimental results. However, many physiological intracellular Ca^2+^ buffers are likely to exhibit strong pH dependence, similarly to EGTA^23, 24^. This is supported by the observations that intracellular alkalinisation induced by application of NH_4_Cl to the bath in Ca^2+^ imaging experiments, when cells have endogenous intracellular Ca^2+^ buffering, potentiates store-operated Ca^2+^ entry in platelets and HT-29 cells^21, 25^. Therefore, results obtained using EGTA, rather than BAPTA, may have more physiological relevance. Much bigger amplitude of I_CRAC_ activated by IP_3_ in the presence of BATPA in the pipette solution, compared to EGTA, was noticed very early on 19. However, the reason for this difference remains poorly understood.

The only residue that has been implicated in I_CRAC_ dependence on pH_i_ so far is His 155 in Orai1^10^. H155F mutation in Orai1 was shown to abolish the increase of I_CRAC_ amplitude in response to intracellular alkalinisation, but I_CRAC_ mediated by H155F-Orai1 was still inhibited by about 60% at low pH_i_^10^, which implies that His 155 is unlikely to be the only site that mediates I_CRAC_ regulation by pH_i_. Data presented in this work indicate that Glut 106 in the Orai1 selectivity centre, which can be protonated from the extracellular side^9^, does not contribute to pH_i_ dependence at all, which also suggests that Orai1 pore is not permeable to protons. Presence of seven negatively charged residues within ID_STIM_ and the fact that neutralisation of three of them, D476, D478, and D479, significantly reduced the FCDI, similarly to acidic pH_i_, made ID_STIM_ a good candidate for the pH_i_ sensor of CRAC channel^18^. The results presented here indicate that ID_STIM_ is not involved in pH_i_ dependence of the I_CRAC_ amplitude, but mutations in ID_STIM_ affect pH_i_ regulation of I_CRAC_ Ca^2+^ dependent gating. The kinetics of I_CRAC_ mediated by Orai1/EE482/483AA-STIM1 or Orai1/DD475/476AA-STIM1 was not appreciably affected by either acidic, or alkaline pH_i_. It is unlikely, however, that protonation/deprotonation of negatively charged resides in ID_STIM_ is responsible for the changes in I_CRAC_ kinetics induced by the changes in pH_i_. Neutralisation of aspartates 482 and 483 increases FCDI, so protonation of these aspartates alone cannot be responsible for reduced FCDI at acidic pH_i_. It has been shown previously that neutralization of these aspartates together with glutamates in ID_STIM_ reduce FCDI^18^, *i*.*e*. the effect of neutralisation of glutamates overcomes the effect of neutralisation of aspartates. This suggests that if glutamates in the ID_STIM_ were protonated at acidic pH_i_, Orai1/EE482/483AA-STIM1 would display dependence of kinetics on pH_i_ similar to that of WT I_CRAC_. However, this was not the case, which excludes ID_STIM_ as a direct pH_i_ sensor.

Interestingly, EE482/483AA-STIM1 significantly diminished the dependence of I_CRAC_ kinetics not only on pH_i_, but also on the relative expression levels of STIM1 and Orai1. This could’ve been a result of saturating levels of expression of the mutant STIM1, compared to Orai1. If the expression levels of STIM1 are very high, moderate changes in the affinity of STIM1 biding to Orai1 due to changes in pH_i_, or moderate increase in Orai1 expression, are unlikely to have an appreciable effect on I_CRAC_ kinetics. However, when the amounts of STIM1 are close to saturating, 2-APB does not potentiate I_CRAC_^16^. Application of 2-APB to Orai1 EE482/483AA-STIM1 mediated I_CRAC_ caused the same level of potentiation as in WT I_CRAC_ at all intracellular pH tested. This indicates that expression levels of mutant STIM1 were not different from that of WT STIM1, and that pH_i_ affected functional coupling of Orai1 with mutant STIM1 in the same way it has affected it’s functional coupling with WT STIM1. The lack of the dependence of Orai1 EE482/483AA-STIM1 I_CRAC_ kinetics on the Orai1:STIM1 relative expression ratio and pH_i_ suggests that the minimum number of this mutant STIM1 peptides which is needed to open Orai1 pore, is sufficient to support fully functional I_CRAC_ FCDI.

In conclusion, the results presented here support the hypothesis that I_CRAC_ inhibition by intracellular acidification is caused by disruption of functional coupling of STIM1 and Orai1, whereas the increase in I_CRAC_ amplitude at alkaline pH_i_ in the presence of EGTA is mainly due to increased Ca^2+^ buffering capacity of EGTA. Negatively charged ID_STIM_ is not a direct pH_i_ sensor, but mutations neutralising negative charges in ID_STIM_ affect pH_i_ dependence of I_CRAC_ kinetics by changing the interaction between STIM1 and Orai1.

## Methods

### Cell culture and transfections

HEK-293T cells [human embryonic kidney-293 cells expressing the large T antigen of SV40 (simian virus 40)] (A.T.C.C. CRL 11268) were cultured at 37 °C in 5% (v/v) CO_2_ in air in DMEM (Dulbecco’s modified Eagle’s medium) supplemented with 100 *μ*M nonessential amino acids, 2 mM L-glutamine and 10% fetal bovine serum^9, 16^. To co-express WT Orai1 with WT STIM1 or double STIM1 mutants (EE482/483AA and DD475/476AA), cells seeded on glass cover slips were transfected using Polyfect (Qiagen) transfection reagent according to the manufacturer’s instructions. The Orai1 and STIM1 (WT or mutant) plasmids were transfected at two Orai1:STIM1 molar ratios 1:1 and 1:4^9, 16^. Plasmids containing EE482/483AA and DD475/476AA double STIM1 mutants were generously provided by Prof Richard Lewis (Stanford University, USA).

### Patch clamping

Whole-cell patch clamping was performed at room temperature (23 °C) using a computer based patch-clamp amplifier (EPC-9, HEKA Elektronik) and PULSE software (HEKA Elektronik) as previously described^9, 16^. The control bath solution contained 140 mM NaCl, 4 mM CsCl, 10 mM CaCl_2_, 2 mM MgCl_2_ and 10 mM HEPES adjusted to pH 7.4 with NaOH. Depletion of intracellular Ca^2+^ stores was achieved using 20 *μ*M Ins(3,4,5)*P*3 (Sigma) added to an internal solution containing 130 mM caesium glutamate, 10 mM CsCl, 5 mM MgCl_2_, 1 mM MgATP, 10 mM EGTA and either 10 mM MES adjusted to pH 6.3 with NaOH, or 10 mM HEPES adjusted to pH 7.3 or 8.3 with NaOH. Patch pipettes were pulled from borosilicate glass and fire polished to give a pipette resistance between 2 and 4 MΩ. Series resistance did not exceed 15 MΩ and was 50–70% compensated. Traces obtained before activation of I_CRAC_, or after its inhibition with 10 µM La^3+^ were used for leakage subtraction.

### Data analysis

To obtain apparent (relative) open probability (*P*_o_) curves of CRAC channels, instantaneous tail currents recorded in response to voltage steps to −100 mV after test pulses between −140 and 80 mV, applied every 5 s in 20 mV increments, were normalised to the amplitude of the instantaneous tail current recorded after test pulse to 80 mV and plotted against corresponding test pulse voltage^9^. The length of the test pulses was set to 150 ms to make sure that both gating processes of I_CRAC_ – inactivation and re-activation are captured in one protocol. Were possible, the data points were fitted with the Boltzmann distribution with an offset of the form:

1 $${P}_{{\rm{o}}}(V)={P}_{min}+(1{\textstyle \text{-}}{P}_{min})/(1+\exp (({V}_{1/2}-V)/k))$$

where *P*_min_ is an offset, *V* is the membrane potential, *V*_1/2_ is the half-maximal activation potential (*V*_1/2_ corresponds to the inflexion point of the *P*_o_ curve) and *k* is the slope factor. However, in many cases apparent *P*_o_ data could not be fitted with Boltzmann distribution and the data points were fitted with a smooth curve using cubic spline procedure in Prizm 6 software.
